# Megestrol treatment in patients with hepatocellular carcinoma

**DOI:** 10.1054/bjoc.2001.2092

**Published:** 2001-11

**Authors:** F Farinati, S Gianni, M De Giorgio, S Fiorentini

**Affiliations:** Department of Surgical and Gastroenterological Sciences, Padua University Policlinic Via Giustiniani 2, Padua, 35128, Italy

**Keywords:** hepatocellular carcinoma, anti-oestrogens, megestrol


British Journal of Cancer (2001) 85(10), 1606–1608
© 2001 Cancer Research Campaign
doi: 10.1054/ bjoc.2001.2092, available online at http://www.idealibrary.com on IDEAL

Letter to the Editor

### Megestrol treatment in patients with hepatocellular carcinoma

**Keywords**: hepatocellular carcinoma; anti-oestrogens; megestrol

We read with great interest the paper by Villa et al (2001) on megestrol treatment of inoperable HCC. Having been involved in hormonal treatment of HCC since the 80ie (Farinati et al, 1990) and knowing that, despite all efforts, there is no effective treatment at present for a patient with advanced disease (Simonetti et al, 1997), any good news on a new treatment is eagerly awaited. With this in mind, and based on previously published papers (Chao et al, 1997) and on personal communications by Professor Villa, we also started in 1999 an open label, uncontrolled prospective trial of megestrol in the treatment of HCC patients. At least at that time performing a second biopsy to test the type of oestrogen receptor present was hardly conceivable and the discussion on whether or not a biopsy is mandatory (and ethical) in patients with cirrhosis and a liver lesion confirmed by both US and CT scanning and by alfafetoprotein levels higher than 200–400 ng/ml was open, as it is now. We therefore decided to enroll all the consecutive patients diagnosed in our unit as having HCC on the bases of either a confirmatory biopsy or a compatible CT scanning and significantly increased alfafetoprotein levels, without testing for oestrogen receptor. To be able to judge on biohumoral and oncological response rate, only patients in whom alfafetoprotein levels had been assessed and were higher that the cut-off (14 ng/ml) and hepatic masses were clearly measurable by US scanning were selected. On these premises, we enrolled 37 consecutive patients (28 males, 9 females, mean age 67.8, range 56–79) with HCC in cirrhosis (23 HCV-related, 5 HBV-related, 7 in alcoholic cirrhosis, 2 with mixed [HCV+HBV] etiology). No patient was eligible for OLTx, surgery, percutaneous locoregional treatments (PEI or RFA), arterial lipiodol-mediated chemoembolization on the basis of the tumour burden, the presence of neoplastic portal thrombosis or their Child-Pugh status (Child-Pugh A = 21, B = 15, C = 1). We decided to administer megestrol (160 mg daily) for at least 60 days, before reassessing the patients' conditions. The drug was withdrawn in case of clear disease progression, patients' death or appearance of serious adverse events clearly correlated with megestrol administration. The mean time of administration was 4.6 months (range 7–395 days). Oncological and biohumoral (alfafetoprotein levels) response rate were assessed together with patients' performance status (Karnowski index).

In one patient treatment was suspended after 1 week because of hospital admission due to development of ascites. Adverse events were observed in 16 patients: 5 ascites, 5 fever, 3 portal thrombosis, 5 itching, 3 bleeding episodes (2 from oesophageal varices and 1 from duodenal ulcer), 5 appearance or worsening of weakness, 1 deep venous thrombosis. The relation of these episodes to megestrol treatment was judged as possible in the large majority of cases and probable with respect to deep venous thrombosis, described during megestrol treatment (Force et al, 1999) and duodenal ulcer bleeding, also reported in the literature (Colleoni et al, 1995). As of 1 May 2001 26 patients are dead and 10 survive, mean survival being 7 months, with a range of 2 to 19 months and a 19% 1-year survival. Overall, one partial response was observed in a female HCC patient with HCV-related cirrhosis (Child A) in whom tumour mass decreased by more than 50%, with a drop of alfafetoprotein levels from 110 ng/dl to 18 ng/dl and with weight gain of 23 kilograms, accompanied by appetite increase, hair growth and psycological improvement (Figure 1). In this patient megestrol was first reduced for development of obesity and then withdrawn for admission due to bleeding from duodenal ulcer (see Figure 1). After suspension and then despite readministration, alfafetoprotein increased and an additional nodule appeared at US scanning. In two other patients a biohumoral response was observed with reduction of alfafetoprotein levels from 1256 ng/dl to 22 ng/dl in the first patient, who had a stable disease from the oncological point of view, and from 2090 ng/dl to 1044 ng/dl in a second patient with, however, disease progression at US. Overall, mean alfafetoprotein levels went from 2640+/-2218 ng/dl to 3093+/–4120 ng/dl at the end of treatment, with a 17% increase. Additionally, 7 out of the 37 patients experienced a slight amelioration of their performance status (Karnowski score from 70 to 80 as a mean). In all the remaining patients (28/37) progressive disease was observed, with no improvement in performance status.

This prospective study is based on a pragmatic approach, which is in any case much closer to the real clinical practice, since very few centres will have the possibility to test liver biopsies from HCC patients for the presence of wild-type or mutated oestrogen receptor, in a situation in which the recent EASL guidelines state that a liver biopsy in a patient with suspected HCC is necessary only in case of lesions of less than 2 cm or in doubtful situations. The results obtained confirm that megestrol can, in

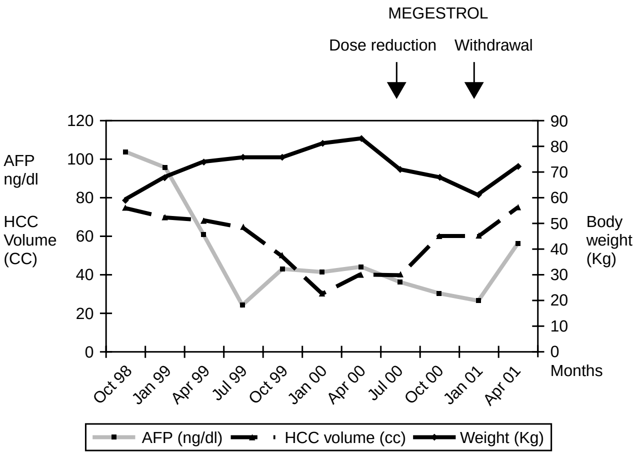

**Figure 1**

*Received 11 June 2001*
*Accepted 26 July 2001*

*Correspondence to:* F Farinati some instances, favourably influence the course of the disease, but that this, in a consecutive series of HCC patients, happens in a small minority of cases, the treatment being purely palliative in a relatively larger sub-group and of no use in the majority. This, since the variant receptor is to be detected in HbsAg positive patients (Villa et al, 1998), may well have something to do with the fact that we, by now, see mostly patients with HCV-related chronic liver damage and HCC. On the other hand, in four patients development of portal or deep venous thrombosis was observed, which could be considered an expected worsening in patients with HCC, with respect to portal thrombosis, but that could have something to do with the pro-coagulant properties of megestrol at least in the case of deep venous damage (Force et al, 1999). Our experience therefore, albeit limited and uncontrolled, suggests that side effects, particularly on blood coagulation, may not be irrelevant.

In summary, our feeling is that megestrol and the variant oestrogen receptor may be more a step in the understanding of the patho-physiological mechanisms underlying HCC development and progression than a true advance in HCC treatment but we are available to change our mind if new additional data will confirm those reported by Villa et al (2001) and deny our findings. This is also because the tamoxifen story has taught everyone, and us more than others, that preliminary, successful small size trials are to be confirmed by large size prospective randomized placebo-controlled studies before a drug enters routine clinical practice.

### ACKNOWLEDGEMENT

Supported in part by a grant from the Italian Ministry of University and Scientific and Technologic Research (MM06174173–003).

**F Farinati, S Gianni, M De Giorgio and S Fiorentini**

*Department of Surgical and Gastroenterological Sciences, Padua University Policlinic Via Giustiniani 2, 35128, Padua, Italy*

### REFERENCE S

Chao Y, Chan WK, Wang SS, Lai KH, Chi CW, Lin CY, Chan A, Whang-Peng J, Lui WY and Lee SD (1997) Phase II study of megestrol acetate in the treatment of hepatocellular carcinoma. *J Gastroenterol Hepatol* **12**(**4**): 277–281

Colleoni M, Nelli P, Vicario G, Mastrpasqua G and Manente P (1995) Megestrol acetate in unresectable hepatocellular carcinoma. *Tumori* **81**(**5**): 351–353

Farinati F, Salvagnini M, De Maria N, Fornasiero A, Chiaramonte M, Rossaro L and Naccarato R (1999) Unresectable hepatocellular carcinoma: a prospective controlled trial with tamoxifen. *J Hepatol* **11**: 297

Force L, Barrufet P, Herrerasa Z and Bolibar I (1999) Deep venous thrombosis and megestrol in patients with HIV infection. *AIDS* **13**(**11**): 1425–1426

Simonetti RG, Liberati A, Angiolini C and Pagliaro L (1997) Treatment of hepatocellular carcinoma: a systematic review of randomized controlled trials. *Ann Oncol* **8**(**2**): 117–136

Villa E, Dugani A, Moles A, Camellini L, Grottola A, Buttafoco P, Merighi A, Ferretti I, Esposito P, Miglioli L, Bagni A, Troisi R, De Hemptinne B, Praet M, Callea F and Manenti F (1998) Variant liver estrogen receptor transcripts already occur at an early stage of chronic liver disease. *Hepatology* **27**(**4**): 983–988

Villa E, Ferretti I, Grottola A, Buttafoco P, Buono MG, Giannini F, Manno M, Bertani H, Dugani A and Manenti F (2001) Hormonal therapy with megestrol in inoperable hepatocellular carcinoma characterized by variant oestrogen receptors. *Br J Cancer* **84**(**7**): 881–885

### Reply

**Sir,**

Dr Farinati and colleagues have reported an uncontrolled experience with megestrol in the treatment of inoperable hepatocellular carcinoma. Their results were somewhat different from those we have reported in *Br J Cancer* (Villa et al, 2001) and we would like to add some considerations.

Despite the fact that megestrol has a rationale in HCC characterised by both wild-type and variant oestrogen receptors (*v*ERs), as its action is displayed at post-receptorial level (and therefore able to interfere both *v*ERs and wild-type ERs (*wt*ERs), still the natural history of HCC with *wt*ERs is so favourable and the growth speed of the tumours so slow that megestrol or any other antihormonal drug, would not add much in terms of amelioration of prognosis (Villa et al, 2000). The choice of treating with megestrol only patients with *v*ERs was therefore justified by the much more aggressive clinical course of these HCCs, which could allow easier identification of any effect on tumour growth or an improvement in survival. As variant ERs are usually not more than 30% of patients with HCC, the higher percentage of patients with wild-type ERs could obscure a favourable effect of megestrol when this treatment is used in a mixed population.

Furthermore, the uncontrolled design of the study by Farinati et al, could not allow perception of the most relevant finding of our study, i.e. the improvement in survival in treated patients. It was, in fact, already evident from our data that megestrol did not determine regression of tumour mass (except in a few cases) whereas slowing down of tumour growth was remarkable in comparison with untreated patients. This effect was short-lived but sufficient to determine a significant improvement of survival at 1 year (Villa et al, 2001). Certainly, megestrol was not powerful enough to cure HCC, but in these patients with ominous prognosis, a gain of 10–12 months in survival can be considered an achievement. In a few of them, clinical improvement was also accompanied by significant regression of tumour mass which allowed performance of radical treatment (E Villa and V Mazzaferro, personal communication).

Last but not least, the side effects reported by Farinati et al (e.g. portal vein thrombosis, deep vein thrombosis, bleeding etc.) may also spontaneously occur in HCC patients: again the uncontrolled experimental design by Farinati was certainly not suitable for correctly allocating side effects to therapy or to disease. Indeed, in our series increase in appetite and in weight occurred in a remarkable percentage of treated patients and in none of the control. However, as for the vascular complications, deep vein thrombosis occurred in the same proportion in treated and untreated patients.

In conclusion, these considerations underline the need to observe very strict methodological rules when performing therapeutic trials: only a controlled method allows identification and correct allocation of both benefits and side effects.

**E Villa, N De Maria, A Colantoni, M Manno and H Bertani**

### REFERENCES

Villa E, Moles A, Ferretti I, Buttafoco P, Grottola A, Del Bvono MG, De Santis M and Manenti F (2000) Natural history of inoperable hepatocellular carcinoma:

